# *Ab Initio* Calculations of Possible γ-Gauche Effects in the ^13^C-NMR for Methine and Carbonyl Carbons in Precise Polyethylene Acrylic Acid Copolymers

**DOI:** 10.3390/molecules18089010

**Published:** 2013-07-29

**Authors:** Todd M. Alam

**Affiliations:** Department of Nanostructured and Electronic Materials, Sandia National Laboratories, Albuquerque, NM 87185, USA; E-Mail: tmalam@sandia.gov; Tel.: +1-505-844-1225; Fax: +1-505-844-2974

**Keywords:** *ab initio*, ^13^C-NMR, chemical shift, *trans-gauche*, γ-*gauche*, methine, carbonyl

## Abstract

The impacts of local polymer chain conformations on the methine and carbonyl ^13^C-NMR chemical shifts for polyethylene acrylic acid p(E-AA) copolymers were predicted using *ab initio* methods. Using small molecular cluster models, the magnitude and sign of the γ-*gauche* torsional angle effect, along with the impact of local tetrahedral structure distortions near the carbonyl group, on the ^13^C-NMR chemical shifts were determined. These ^13^C-NMR chemical shift variations were compared to the experimental trends observed for precise p(E-AA) copolymers as a function acid group spacing and degree of zinc-neutralization in the corresponding p(E-AA) ionomers. These *ab initio* calculations address the future ability of ^13^C-NMR chemical shift variations to provide information about the local chain conformations in p(E-AA) copolymer materials.

## 1. Introduction

In polyethylene (PE) the ^13^C-NMR chemical shift difference between the crystalline and amorphous phases are commonly attributed to the *γ-gauche* effect (*trans-gauche* effect) arising from differences in the local polymer chain conformation [1,2,3,4]. In polymers, intermolecular effects that include chain packing, polar interactions, and hydrogen bonding, can also impact the observed ^13^C-NMR chemical shifts. The γ-*gauche* effect has recently been employed in measuring the backbone and side chain conformations of proteins [5,6,7,8]. *Ab initio* calculations of NMR chemical shifts in amorphous and crystalline polymers and proteins have proven indispensable in determining the local chain conformations and microstructure for these materials. The γ-*gauche* effect has been documented for a variety of substituted PE polymers. For example, the ^13^C-NMR chemical shifts of methylene (CH_2_) segments in substituted vinyl polymers such as polypropylene (PP), polyisobutylene (PIB), poly(vinyl chloride) (PVC) and polybutadiene (PBD) have been thoroughly investigated using *ab initio* methods [9,10,11,12,13]. Previous studies have concentrated on understanding the ^13^C-NMR chemical shift and resonance band shape of the methylene (CH_2_) carbon segment in terms of polymer chain conformations, and the role different functional groups have on the micro-structure. In PVC and PP there have also been limited evaluations of the methine (CH) ^13^C-NMR chemical shifts that demonstrate a γ-*gauche* effect for this carbon [1,2,9,12]. 

**Scheme 1:**
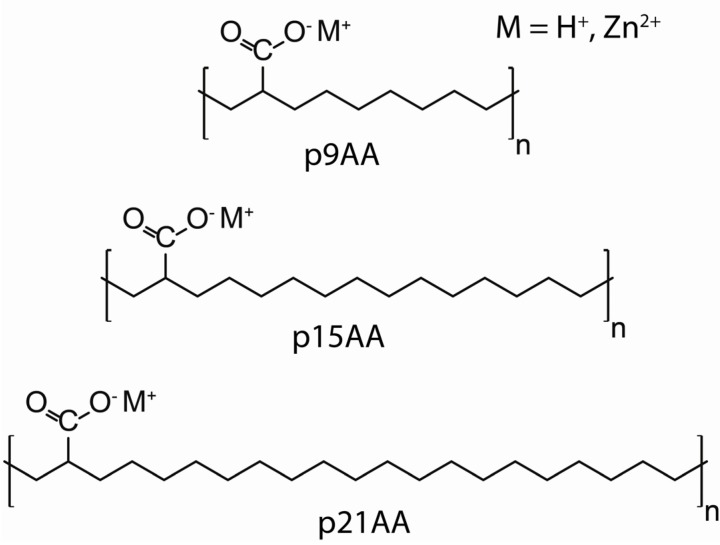
Precise p(E-AA) Copolymers and Ionomers.

Recently our group reported the ^13^C-MAS-NMR for a series of precise polyethylene acrylic acid p(E-AA) copolymers and associated Zn-substituted ionomers [14]. These polymers have a carboxylic acid substituent located at precise locations along the PE backbone chain, and were used to produce the corresponding Zn-neutralized ionomers (Scheme 1).

The methylene (CH_2_) ^13^C-NMR chemical shifts were used to identify and quantify the crystalline and amorphous phases present in these p(E-AA) copolymers, with the spectral identification based on the well-established γ*-gauche* effect in PE type polymers. The broad resonance corresponding to the amorphous *trans/gauche* phase is shifted approximately −2 ppm with respect to the all *trans* conformation in the crystalline phase [14]. For these same precise p(E-AA) copolymers distinct trends in the ^13^C-NMR chemical shifts were also observed for the methine (CH) carbon as a function of the chain length spacing between the carboxylic acid groups, and as a function of the extent of Zn-neutralization. For example, the chemical shift of the CH resonance increases by almost +3 ppm when the chain space length varies from p9AA to p21AA. In addition, there are distinct multiple CH resonances observed in the p9AA copolymer (δ = +44.5 and +46.5 ppm) and the corresponding Zn-neutralized ionomer (δ = +47.0 and 49.6 ppm). The carbonyl (CO) ^13^C-NMR chemical shift revealed only a minor chemical shift variation, increasing +0.7 ppm between the p9AA and p21AA copolymers. Zn neutralization produced a larger variation, increasing the shift between +2 and +4 ppm [14]. Since these precise copolymers have isolated functional groups, investigation of the NMR chemical shift for the CH carbon is proposed to provide a direct probe for the local chain conformation at the point of chain functionalization.

In our original analysis of these ^13^C-NMR chemical shift trends in the p(E-AA) copolymers and ionomers, we argued that the CH variations resulted from a *γ-gauche* effect, and that Zn-neutralization did not have a large influence on the observed shift. In contrast, we suggested that the observed CO ^13^C-NMR chemical shift variations were dominated by the zinc neutralization of the acid group, with the *γ-gauche* effect playing a minor role. Unfortunately, there are a limited number of studies establishing the magnitude of the *γ-gauche* effect for CH and CO carbons in amorphous polymer systems, in particular, systems involving carboxylic acid pendant groups. In addition, the small 2-ethyl-butanoic acid model cluster employed to initially model the ^13^C-NMR chemical shifts using *ab initio* methods was too small to eliminate end-group effects from impacting the calculated shifts of the central CH, and only a very limited number of chain conformations were considered in that analysis [14]. 

This paper addresses these issues by presenting a series of *ab initio* NMR ^13^C-NMR chemical shift calculations using a larger molecular cluster to model the acrylic acid polymer fragment as a function of multiple torsional angles describing the chain conformation. The magnitude and sign of the *γ-gauche* effect were evaluated for both the CH and CO carbons, and are compared to the ^13^C-NMR experimental results observed in the p(E-AA) copolymers and Zn-neutralized ionomers [14].

## 2. Results and Discussion

**Figure 1:**
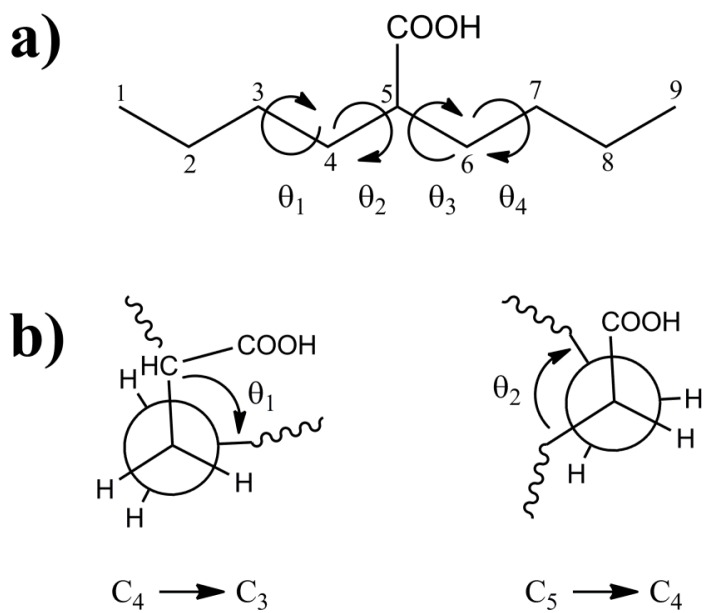
(**a**) The model 2-butylhexanoic acid model cluster with the carbon numbering and torsional dihedral angles defined. (**b**) Projections along the C_4_-C_3_ and C_5_-C_4_ bonds defining the θ_1_ and θ_2_ dihedral angles.

The 2-butylhexanoic acid molecular fragment (Figure 1) was chosen as the model cluster for evaluation of the CH and CO ^13^C-NMR chemical shifts within p(E-AA) copolymers. The methine (CH) carbon, C5, is sufficiently removed from the end of this molecular cluster to minimize end-group effects [9]. The dihedral angles (θ_1_, θ_2_, θ_3_, θ_4_) describing the local polymer chain conformations around the CH carbon ranging from −180° < θ*_i_* ≤ 180° are shown in Figure 1a. The rotational sense of the individual dihedral angles was always defined looking down the bond originating at the carbon closest to the central COOH pendant group (*i.e.*, along the C_4_-C_3_ for θ_1_ or the C_5_-C_4_ bond for θ_2_) as depicted in Figure 1b. The rotations of dihedral angles near the ends of the chains were not considered to be important in describing the local conformation for the central carbon, such that these dihedral angles were not constrained and allowed to adopt a minimum energy conformation.

**Figure 2:**
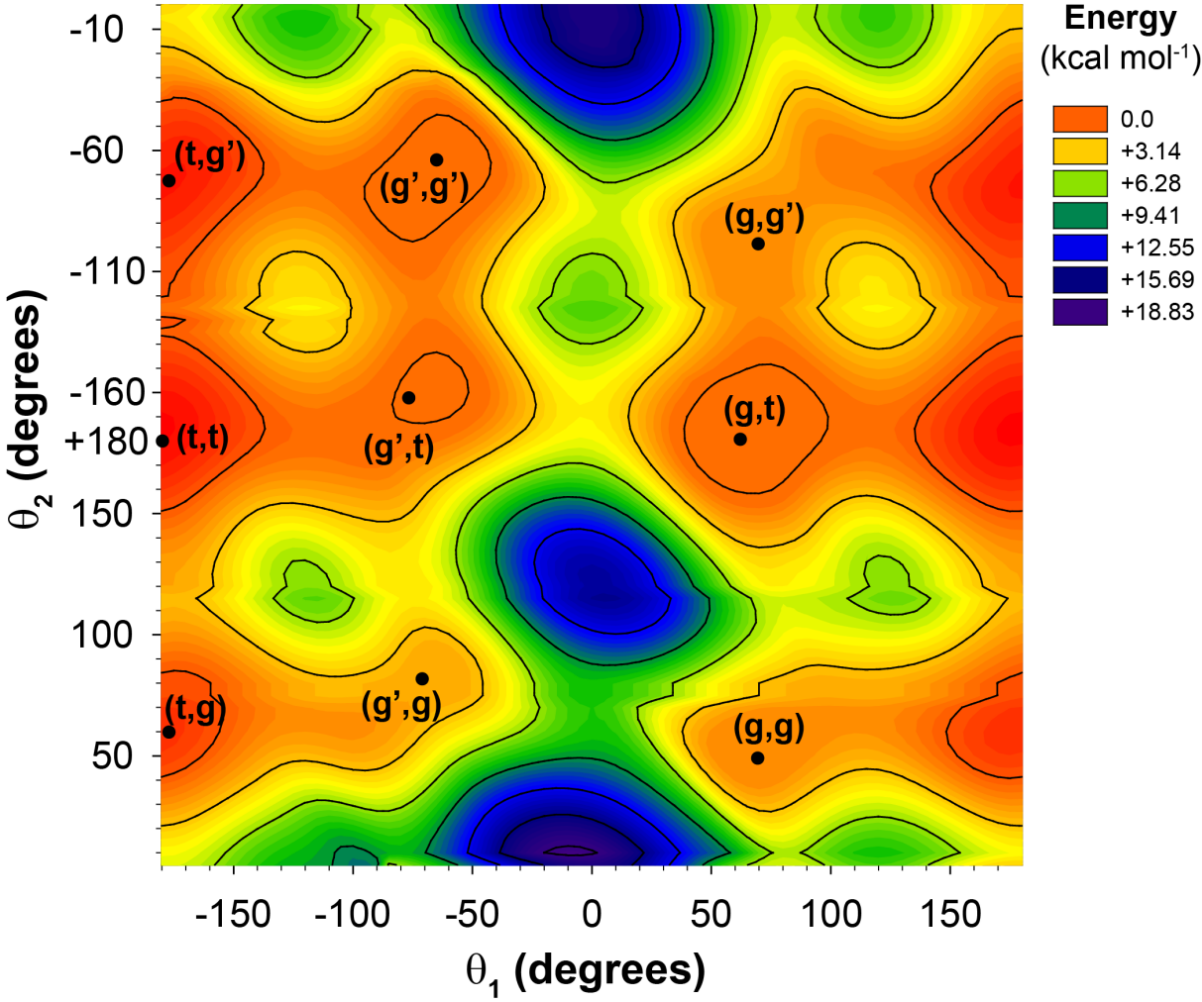
The potential energy surface for the 2-butylhexanoic acid model cluster as a function of the θ_1_ and θ_2_ dihedral angles.

The potential energy surface as a function of the dihedral rotation angles was calculated in the gas phase for the model cluster. Step-wise rotation of a single dihedral angle (while allowing all other structural constraints to optimize) gives rise to the classic potential energy surface with three local energy minima designated as *trans* (t), along with two *gauche* (g and g’) conformations. The potential energy surface as a function of rotation about both the θ_1_ and θ_2_ dihedral angles reveals 9 local minima as revealed in Figure 2. The angles and relative energies for these 9 minima are given in Table 1.

The potential energy surface as a function of the θ_3_ and θ_4_ dihedral angles is identical to the θ_1_ and θ_2_ surface (Figure 2) except for a change in the sense of rotation in defining the g and g’ conformations. The potential energy surface involving all four dihedral angles (θ_1_, θ_2_, θ_3_, θ_4_) is therefore expected to contain 81 different local minima. The structures of the model clusters corresponding to these local energy minima were extracted and used to model the dominant polymer chain conformations. It should be noted that these are gas phase calculations for a short model fragment, and that the minima and relative populations obtained are unlikely to fully reflect the conformations present within polymers. It has previously been demonstrated that the size of the molecular model fragment can be restricted to γ- and δ- neighbors for the carbon in question, with only minor changes in the predicted chemical shifts resulting from the longer range end-effects [9]. 

**Table 1:** The dihedral angles, relative potential energies and populations for the local minima obtained from the θ_1_, θ_2_ potential energy surface.

Conformation (θ_1_, θ_2_)	θ_1_ (deg)	θ_2_ (deg)	ΔE (kcal/mol)	*Fractional Population, p^i^*
tg'	−175	−73	0.69	0.145
tt	+180	+180	0.00	0.461
tg	−175	+60	1.01	0.084
g'g'	−65	−70	1.32	0.049
g't	−68	−160	1.26	0.055
g'g	−65	+80	3.36	0.002
gg'	+65	−100	3.07	0.003
gt	+62	+180	0.50	0.198
gg	+68	+52	3.00	0.003

### 2.1. Methine ^13^C-NMR Chemical Shifts

**Figure 3:**
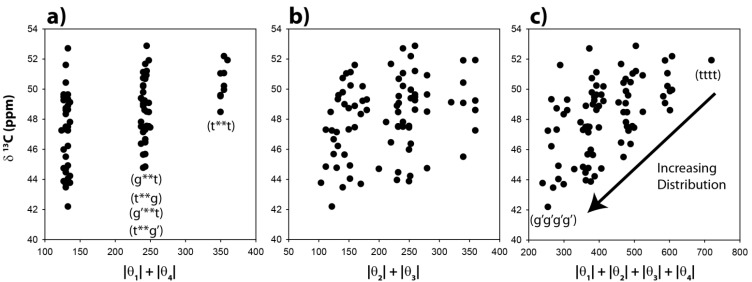
Predicted ^13^C-NMR chemical shift variation of the methine (CH) carbon as a function of (**a**) the |θ|_1_ + |θ|_4_ dihedral angle summation, (**b**) the |θ|_2_ + |θ|_3_ dihedral angle summation, and (**c**) the |θ|_1_ + |θ|_2_ + |θ|_3_ + |θ|_4_ dihedral angle summation.

The ^13^C-NMR chemical shifts for the CH carbons in these 81 optimized clusters were evaluated and are shown as a function of the |θ|_1_ + |θ|_4_ angle summation in Figure 3a. Variations of these dihedral angles describe the relative γ orientation of the CH carbon with respect to the remainder of the polymer chain on each side. For conformations where these two dihedral angles are both in a *trans* conformation the |θ|_1_ + |θ|_4_ summation will be 360°, while those conformations involving both dihedrals in a *gauche* orientation will have a summation of ~120°. It is immediately apparent that the calculated ^13^C-NMR chemical shifts show significant variations, and are not a simple function of the dihedral angles. For the θ_1_, θ_4_ *gauche-gauche* conformations (g**g, g**g’, g’**g, g’**g’, where * denotes the unspecified θ_2_ and θ_3_ dihedral angles) there is an almost 10 ppm chemical shift variation observed (vertical distribution). Not all of these chain conformations are energetically favorable, and would not be necessarily observable. For example, the relative energy of the gggg chain conformation is nearly 9 kcal higher than the tttt conformation. The all-*gauss* conformations (gggg, gggg’, ggg’g’ *etc.*) where found to have very high relative energies, and are not expected to contribute significantly to the observed ^13^C-NMR spectra. 

Obviously the γ-*gauche* effect for the CH carbon, as controlled by the conformation of the θ_1_ and θ_4_ dihedral angles, is not the dominating (or only) factor dictating the ^13^C-NMR chemical shift, and that other structural components of the polymer chain are also influencing the chemical variations. These results are very different than those predicted for the CH_2_ carbon in PE, where the γ-*gauche* effect is distinct and on the order of Δδ ~ −4 ppm in going from all-*trans* to all-*gauche* in the γ carbon position [12]. In addition, the broad distribution of predicted CH ^13^C-NMR chemical shifts was not observed experimentally, where relatively narrow line widths were observed for the CH carbon in all p(E-AA) copolymer materials [14]. It is important to note that values of the θ_1_ and θ_4_ dihedral angles reveal a relatively narrow angle distribution (Figure 3a, horizontal spread) demonstrating that the potential energy surface for this dihedral angle pair was not significantly perturbed by the presence of the COOH group attached to C_5_. 

The predicted CH ^13^C-NMR chemical shift as a function of the |θ|_2_ + |θ|_3_ dihedral angle summation is shown in Figure 3b, and again reveals no correlation with these dihedral angles. It is clear that distinct β-orientational effects are minimal for the CH carbon. There is a significant variation (horizontal spread) in the θ_2_ and θ_3_ angles observed (Figure 3b), demonstrating that the potential energy surface of these dihedral angles was perturbed from classic *trans-gauche* values (180°, 60°) observed in un-substituted PE. The ^13^C NMR chemical shift as a function of all four dihedral angles is shown in Figure 3c. No clear correlations are observed, but it can be argued that there is a decrease in the average chemical shift, and an increase in the distribution of chemical shifts with respect to the all *trans* conformation (tttt) when higher percentages of the *gauche* conformation are introduced into the polymer chain. These results demonstrate that while there is some influence of the *γ-gauche* on the CH ^13^C NMR chemical shift, there are clearly other structural perturbations influencing the shift.

**Figure 4:**
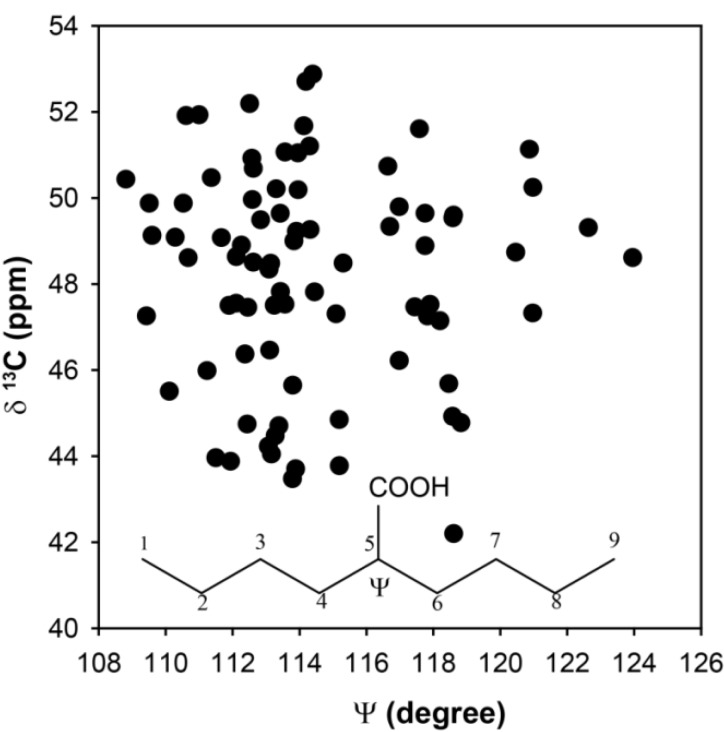
Predicted ^13^C-NMR chemical shift variation for the methine (CH) carbon as a function of the bond angle Ψ.

In previous *ab initio* investigation of ^13^C-NMR chemical shifts in “crowded” polymer systems such as PIB, there were deviations from the ideal tetrahedral coordination underlying the geometric assumptions in formulating the γ-*gauche* effect. As the size of the substituent was increased, the distortions of the chain tetrahedral angles near the substituent were perturbed. In the current study, the tetrahedral angles were not constrained such that these types of structural perturbations could occur in the selected clusters. To explore if tetrahedral distortions might account for the observed chemical shift distribution, the CH ^13^C-NMR chemical shift as a function of the C_4_-C_5_-C_6_ bond angle Ψ are shown in Figure 4. While there is a range of dihedrals observed, the distribution of the chemical shifts is again significant (Δδ ~ 10 ppm), but does not appear to have a simple correlation with bond angle distortions. Based on these *ab initio* results there does not appear to be a single structural parameter that can be extracted from the experimentally determined ^13^C-NMR chemical shifts in the p(E-AA) copolymers. 

These CH chemical shift correlations (Figure 3) along with the experimentally narrow CH resonance argue that only a limited number of local chain conformations occur around the central CH species in these p(E-AA) copolymers and corresponding Zn-modified ionomers. Other possibilities include the partitioning of CH defect between the crystalline and amorphous phases within the p(E-AA) copolymers, or that local dynamics are averaging the ^13^C chemical shift over multiple conformations. The p21AA sample contained ~ 44% crystalline *trans* conformation phase, and the remainder of the material was in the amorphous *trans*/*gauche* or *gauche* defect phase, while the p9AA sample is entirely in an amorphous *trans/gauche* phase. For the p21AA polymer only a single CH resonance was observed, inconsistent with the CH defect being present in both the crystalline and amorphous phases (*i.e.*, partitioned into a single phase). On the other hand, the reduction in the CH ^13^C- NMR chemical shift between the p21AA and p9AA copolymer is consistent with the increase in *gauche* conformations with decreasing chain length. As noted above, the narrow CH line width experimentally observed is at odds with the large chemical shift distributions predicted in Figure 3. The reduced line width may reflect chemical shift averaging due to chain dynamics. A Boltzmann average of the predicted CH ^13^C-NMR chemical shift for the 81 different structures investigated is δ = +50.4 ppm, which is lower than the δ = +51.9 ppm predicted for the all-*trans* conformation (Δδ ~ −1.5 ppm). This chemical shift difference is similar to the change observed experimentally. Chain dynamics have been observed within these p(E-AA) copolymers [14] and in precise methyl-substituted PE [15], based on experimental observation of CH_2_ chemical shift anisotropy (CSA) tensor averaging.

### 2.2. Carbonyl ^13^C-NMR Chemical Shifts

**Figure 5:**
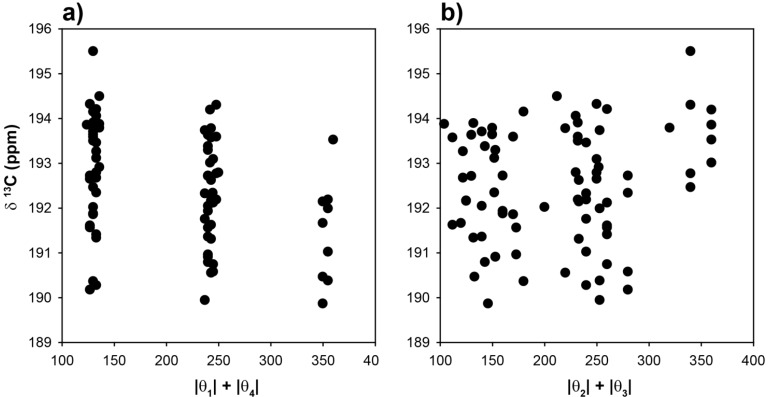
Predicted ^13^C-NMR chemical shift variation of the carbonyl (COOH) carbon as a function of (**a**) the |θ|_1_ + |θ|_4_ dihedral angle summation and, (**b**) the |θ|_2_ + |θ|_3_ dihedral angle summation.

To explore the role of chain conformations on the carbonyl ^13^C-NMR spectra, the COOH chemical shifts for the 81 different clusters were calculated and are shown in Figure 5 as a function of the |θ_1_| + |θ_4_| and |θ_2_| + |θ_3_| dihedral angle summations. In this case the dihedral angles θ_2_ and θ_3_ would control the *γ-gauche* effect. Similar to the methine chemical shifts there is a very large variation of ~15 ppm observed for the COOH chemical shift. This is significantly larger than the experimentally observed variation in the copolymer (~+0.7 ppm), or the Zn-neutralized ionomer (~ +2 to +4 ppm). These results (Figure 5) demonstrate that COOH NMR chemical shift correlations with dihedral angles describing the different chain conformations were not beneficial in determining the local chain conformation. In addition, there was no correlation between the COOH chemical shift and the tetrahedral angle Ψ (data not shown). It was noted that the absolute value of the COOH chemical shifts were very sensitive to the basis set used in structural optimization, but the overall Δδ range as a function of angle was consistent between different levels of theory. This basis set effect reflects the need to adequately model the electron density in the carbonyl region, and required both diffuse and polarized basis functions. These results support the earlier conclusion that for the experimentally observed carbonyl ^13^C-NMR chemical shifts, the γ-*gauche* effects was not observed. It is proposed that the experimental COOH NMR chemical shifts are governed by intermolecular effects such as hydrogen bonding and neutralization. Based on these correlations (Figure 5), the lack of large dispersions in the COOH experimental line width (broadened line shape) argues against multiple different chain conformations in both the p(E-AA) copolymers and the Z,-modified ionomers.

## 3. Computational Details

The potential energy surfaces for the 2-butylhexanoic acid model clusters as a function of the two dihedral angles θ_1_ and θ_2_ (see Figure 1 for definition) were obtained using the Gaussian W09 software (Gaussian Inc., Wallingford, CT, USA) with a 6-311G basis set, density functional theory (DFT) utilizing Becke’s three parameter exchange functional, and the LYP correlation function (B3LYP). The two torsional angles were allowed to vary in 3 degree increments, while the remaining portions of the molecules were allowed to optimize freely. Since there were no restrictions of the θ_3_ and θ_4_ dihedral angles (Figure 1) that portion of the cluster was typically in an extended all-*trans* chain conformation. From this potential energy surface, nine local minima energy conformations were identified (Table 1). Based on symmetry arguments these conformations provided the input for the 81 (9 × 9) clusters used to explore the different θ_1_, θ_2_, θ_3_, θ_4_ conformations describing the polymer chain structure near the methine (CH) and carbonyl (COOH) region. For each θ_1_, θ_2_, θ_3_, θ_4_ conformation (tttt, tttg, *etc.*) the θ_1_, θ_2_, θ_3_, θ_4_ dihedrals where fixed to the local minima values obtained in the simple 2D potential energy scan, while the remaining portions of the molecular cluster were allowed to freely optimize. 

These optimized structures where then used to calculate the ^13^C-NMR shielding tensor (σ) in the Gaussian 09W program with a 6-311G++(2p,2d) basis set using the gauge-including atomic orbital (GIAO) method at the DFT level. The NMR chemical shift for conformation *i* was defined with respect to the isotropic component of the chemical shielding in the TMS reference defined by:

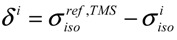
(1)
where positive δ values represent environments that are deshielded and resonate at a higher frequency. The shielding value of σ = +182.47 ppm for TMS was used for ^13^C chemical shift referencing. 

## 4. Conclusions

*Ab initio* calculations were used to explore the polymer chain conformational effects on the methine (CH) and carbonyl (COOH) ^13^C-NMR chemical shifts in polyethylene acrylic acid copolymers. These calculations demonstrate that the observed chemical shift variations could not be directly correlated to the γ-*gauche* effect for either the CH or the COOH carbons. The calculated ^13^C-NMR chemical shifts in the p(E-AA) copolymers behave like other “crowded” substituted polymer systems, where the substituents can give rise to distortions in the local chain structure, precluding some of the structural assumptions underlying the *γ-gauche* effect. The calculations show an increase in the distribution or range of CH chemical shift with the addition of *gauche* chain conformations in the polymer chain, but there is no clear trend that can be assigned to the γ-*gauche* effect. Experimentally, materials containing a significant crystalline component having a high degree of *trans* chain conformations result in increased chemical shift values. On the other hand, the large distribution of chemical shifts predicted as a function of the θ_1_, θ_2_, θ_3_, θ_4_ dihedral angles are not reflected experimentally as broad line widths for the CH carbon, arguing that such distributions of local chain conformations are not present in the precise p(E-AA) copolymers and ionomers, or that there is motional averaging of the chemical shift in the amorphous regions of the p(E-AA) copolymer. Averaging over the different structural minima predicts changes in the CH ^13^C-NMR chemical shift that are similar to those observed experimentally. Unfortunately, these computational results suggest that changes in the CH and COOH ^13^C-NMR chemical shifts do not provide much insight into the local chain conformations for these p(E-AA) materials. 

## References

[1] Tonelli A.E., Schilling F.C. (1981). ^13^C-NMR chemical shifts and microstructure of polymers. Acc. Chem. Res..

[2] Tonelli A.E. (1989). NMR Spectroscopy and Polymer Microstructure: The Conformational Connection.

[3] Grant D.M., Paul E.G. (1964). Carbon-13 magnetic resonance. II chemical shift data for the alkanes. J. Am. Chem. Soc..

[4] Barfield M. (1995). Ab initio IGLO studies of the conformation and substituent dependencies of α-, β-, γ- and δ-effects in the ^13^C NMR spectra of 1-substituted butanes. J. Am. Chem. Soc..

[5] London R.E., Wingad B.D., Mueller G.A. (2008). Dependence of amino acid side chain ^13^C shifts in dihedral angle: Application to conformational analysis. J. Am. Chem. Soc..

[6] Kjaergaard M., Iesmantavicius V., Poulsen F.M. (2011). The interplay between transient α-Helix formation and side chain totamer distributions in disordered proteins probed by methyl chemical shifts. Protein Science.

[7] Hansen D.F., Kay L.E. (2011). Determing valine side-chain totamer conformations in proteins fom methyl ^13^C chemical shifts: Application to the 360 kDa half-proteasome. J. Am. Chem. Soc..

[8] Mulder F.A. A., Filatov M. (2009). NMR chemical shift data and *ab Initio* shielding calculations: Emerging tools for protein structure determination. Chem. Soc. Rev..

[9] Born R., Spiess H.W. (1997). In Ab Initio Calculations of Conformational Effects on ^13^C NMR Spectra of Amorphous Polymers.

[10] Barfield M., Yamamura S.H. (1990). Ab Initio IGLO studies of the conformational dependencies of α-,β-, and γ-Substituent effects in the ^13^C NMR spectra of aliphatic and alicyclic hydrocarbons. J. Am. Chem. Soc..

[11] Born R., Spiess H.W., Kutzelnigg W., Fleischer U., Schindler M. (1994). Conformational effects on ^13^C-NMR chemical shifts of an amorphous polymer: An *ab Initio* Study by the IGLO Method. Macromolecules.

[12] Born R., Spiess H.W. (1995). Conformational effects and configurational splittings in ^13^C NMR spectra of synthetic polymers as investigated by ab Initio individual gauges for localized molecular orbitals (IGLO) calculations. Macromolecules.

[13] Auriemma F., Born R., Spiess H.W., Rosa C.D. (1995). Corradini solid-state ^13^C-NMR investigation of disorder in crystalline syndiotactic polypropylene. Macromolecules.

[14] Jenkins J.E., Seitz M.E., Buitrago C.F., Winey K.I., Opper K.L., Baughman T.W., Wagener K.B., Alam T.M. (2012). The impact of zinc neutralization on the structure and dynamics of precise polyethylene acrylic acid ionomers: A solid-state ^13^C NMR study. Polymer.

[15] Wei Y., Graf R., Sworen J.C., Cheng C.-Y., Bowers C.R., Wagener K.B., Spiess H.W. (2009). Local and collective motions in precise polyolefins with alkyl branches: A combination of ^2^H and ^13^C solid-state NMR spectroscopy. Angew. Chem. Int. Ed..

